# Detection and Anti‐Detection with Microwave‐Infrared Compatible Camouflage Using Asymmetric Composite Metasurface

**DOI:** 10.1002/advs.202410364

**Published:** 2024-09-24

**Authors:** Yanzhao Wang, Huiling Luo, Yanzhang Shao, Hui Wang, Tong Liu, Zhengjie Wang, Kai‐yue Liu, Xiaogang Su, He‐Xiu Xu

**Affiliations:** ^1^ Air and Missile Defense College Air Force Engineering University Xi'an 710051 China; ^2^ Information Engineering University Zhengzhou 450001 China; ^3^ Key Laboratory of Functional Nanocomposites of Shanxi Province School of Materials Science and Engineering North University of China Taiyuan 030051 China

**Keywords:** absorption, asymmetric metasurface, low emission, multispectral camouflage, wavefront control

## Abstract

Detection and anti‐detection with multispectral camouflage are of pivotal importance, while suffer from significant challenges due to the inherent contradiction between detection and anti‐detection and conflict microwave and infrared (IR) stealth mechanisms. Here, a strategy is proposed to asymmetrically control transmitted microwave wavefront under radar‐IR bi‐stealth scheme using composite metasurface. It is engineered composed of infrared stealth layer (IRSL), microwave absorbing layer (MAL), and asymmetric microwave transmissive structure (AMTS) with polarization conversion from top to bottom. Therein, IR emissivity, microwave reflectivity, and transmissivity are simultaneously modulated by elaborately designing the filling ratio of ITO square patches on IRSL, which ensures both efficient microwave transmission and IR camouflage. Furthermore, full‐polarized backward microwave stealth is achieved on MAL by transmitting and absorbing microwaves under *x*‐ and *y*‐ polarization, respectively, while forward wavefront is controlled by precise curvature phase compensation on AMTS according to ray‐tracing technology. For verification, a proof‐of‐concept metadevice is numerically and experimentally characterized. Both results coincide well, demonstrating spiral detective wavefront manipulation under *y*‐polarized forward wave excitation while effective reduction of radar cross section within 8–18 GHz and low IR emissivity (<0.3) for backward detection. This strategy provides a new paradigm for integration of detection and anti‐detection with multispectral camouflage.

## Graphical abstract

This paper proposes a strategy to asymmetrically control transmitted microwave wavefront under microwave‐IR compatible camouflage scheme using composite metasurface. For verification, a proof‐of‐concept metadevice is numerically and experimentally characterized, demonstrating spiral detective wavefront manipulation, RCS reduction, and low IR emissivity for backward detection, which provides a new paradigm for the integration of detection and anti‐detection with multispectral camouflage.

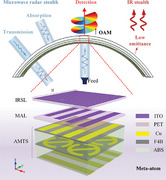

## 1 Introduction

With rapid development of detection technology, traditional mono‐function stealth in a specific spectral band is difficult to fulfill with sharply increased complexity in multispectral comprehensive detection, thus posing a significant threat to military equipment.^[^ ^1^ ^]^ In modern reconnaissance system, microwave and infrared (IR) compatible camouflage are particularly important and challenging due to their inherently contradictory working mechanisms.^[^ ^2^ ^]^ For radar stealth, low reflection is required by employing absorption materials,^[^ ^3^, ^4^, ^5^ ^]^ metamaterial absorbers (MAs)^[^ ^6^, ^7^, ^8^ ^]^ and diffusive metasurfaces^[^ ^9^, ^10^, ^11^ ^]^ to avoid microwave detection. On the contrary, high reflection and low absorption are necessary to avoid the detection of IR detectors, which means low IR emissivity according to Kirchhoff's law.^[^ ^12^, ^13^, ^14^, ^15^ ^]^ To date, the most promising materials for IR stealth include photonic crystals, doped semiconductors, and phase change materials. Therefore, current mainstream radar‐IR bi‐stealth techniques mainly fall into two categories. One approach is to develop a kind of composite doping material that exhibits microwave absorption and low IR emission simultaneously.^[^ ^16^, ^17^, ^18^, ^19^ ^]^ The other is to design a composite structure by covering a microwave absorber with an IR shielding layer (IRSL).^[^ ^20^, ^21^, ^22^ ^]^ Although adjusting material doping concentration and coating thickness achieves elegant compatibility in former strategy, the fabrication process is complicated, particularly in accurately controlling the doping ratio and coating thickness. Furthermore, achieving large‐scale and cost‐effective production of such multispectral camouflage materials remains a significant obstacle owing to a difference of several orders of magnitude in structure.

In harsh warfare environment, a comprehensive effective communication/detection system with multispectral camouflage are crucial when encountering multi‐dimension reconnaissance. Recently, metasurfaces have offered a new approach to multispectral compatible stealth^[^ ^23^, ^24^, ^25^ ^]^ and multifunctional devices^[^ ^26^, ^27^, ^28^, ^29^, ^30^, ^31^, ^32^, ^33^ ^]^ due to their advantages of super thin, easy integration, readily conformal to arbitrary platform, and flexible manipulation of multispectral waves. The key to achieving efficient compatible camouflage lies in applying an IRSL with microwave‐transparent property on a microwave absorber.^[^ ^34^, ^35^, ^36^, ^37^, ^38^, ^39^, ^40^, ^41^, ^42^ ^]^ Among them, low IR emissivity is achieved by utilizing periodic metal grids on IRSL,^[^ ^37^, ^38^, ^39^ ^]^ and a continuous metal ground is usually used for radar absorption.^[^ ^40^, ^41^, ^42^ ^]^ Although the gaps between grids allows microwaves to pass through, the mutual influence of the parametric design on properties within IR and microwave bands is illusive. Moreover, such architecture is not applicable to a specialized practical scenario where transmissive communication/detection and anti‐detection is a concern. Fortunately, the Janus metasurface is a promising candidate for distinct versatile functions when electromagnetic (EM) waves are incident from opposite directions.^[^ ^43^, ^44^, ^45^, ^46^, ^47^, ^48^ ^]^ However, most above attempts are performed for planar architecture, which may seriously hinder the real‐world applications. Although amplitude variations are mainly concerned for flexible metasurfaces due to deformation, phase compensation under arbitrary shapes are neglected.^[^ ^49^, ^50^, ^51^ ^]^ This is especially true for a complicated practical scenario where both microwave‐IR bi‐stealth and wavefront–control detection is a concern. To the best of our knowledge, concurrent multispectral camouflage and transmissive wavefront control based on phase compensation in a curved architecture is rarely reported, whose advantages and advancement will be qualitatively analyzed later.

**Figure 1:**
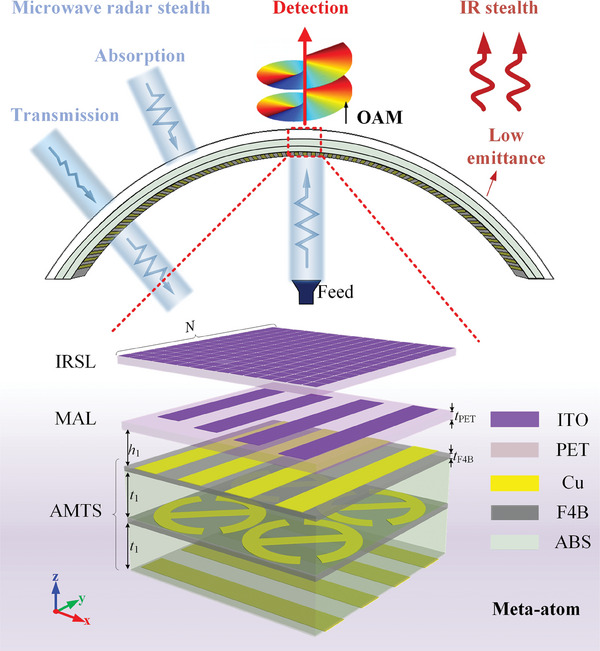
Schematic illustration of asymmetric composite metasurface for detective wavefront control and radar‐IR compatible camouflage (top panel) and the layout of magnified meta‐atom (bottom panel). It is engineered composed of IRSL, MAL, and AMTS from top to bottom. When detected by backward multispectral EM waves along ‐z axis, IR light will be mostly reflected with low IR emittance provided by ITO square patches on IRSL. For microwave radar stealth, *y*‐polarized microwaves are absorbed by ITO strip arrays on MAL after transmitting through IRSL, while *x*‐polarized microwaves are converted to *y*‐polarized wave by AMTS after passing through IRSL and MAL, thereby achieving full‐polarized radar stealth. For forward microwave detection, spiral vortex carrying orbital angular momentum (OAM) is generated by changing quasi‐I‐shape resonators of AMTS when fed by *y*‐polarized horns from the bottom.

Here, we propose a strategy to achieve asymmetric multifunctional metasurface with microwave‐IR compatible camouflage (anti‐detection) and detective wavefront control in conformal scenarios, see the bottom panel in **Figure** **1**. The asymmetric composite metasurface consists of an IRSL, a microwave absorbing layer (MAL), and a triple‐layer asymmetric microwave transmissive structure (AMTS) from top to bottom. The IRSL is constituted by periodic arrays of indium tin oxide (ITO) square patches with high filling ratio, which provides low IR emissivity and meanwhile transmits microwaves with high efficiency. Moreover, IR emissivity, microwave reflectivity, and transmissivity can be simultaneously modulated by elaborately designing the filling ratio of ITO materials. Full‐polarized anti‐detection is achieved through integration of MAL and AMTS. For *y*‐polarized backward microwaves incident along ‐z axis, MAL consisting of ITO strip arrays and AMTS composed of orthogonal gratings and quasi‐I‐shape resonators functioning as a metal ground forms a sandwiched absorption structure for microwave stealth. For *x*‐polarized backward wave detection, AMTS functions as a frequency‐selective spacer filter and thus allows the wave transmit through with a transmission window. Furthermore, phase control is achieved by changing quasi‐I‐shape resonators of AMTS when fed by *y*‐polarized horns from the bottom. Therein, on demand microwave detection enabled by desired manipulation of forward transmission wavefront is engineered through precise phase compensation in curved architecture according to ray‐tracing method. As a proof‐of‐concept demonstration, a spiral vortex beam generator with microwave‐IR compatible camouflage was fabricated and characterized on a cylindrical platform. The proposed metadevice preserves more than 10 dB RCS reduction across 8–18 GHz (covering X and Ku bands) and simultaneously generates a pre‐defined forward vortex beam. Furthermore, it demonstrates a low IR emissivity (<0.3) within both 3–5 and 8–14 µm, fulfilling the requirement of IR stealth. Our strategy will enhance multispectral compatible stealth capabilities to avoid diverse detection modes, and is anticipated to enhance military reconnaissance in harsh warfare environments.

## 2 Concept and Design

To begin with, we first elaborate on our strategy of detective wavefront control and anti‐detection with multispectral compatible camouflage, as depicted in Figure 1. To evade backward multispectral detection, IR camouflage is achieved on IRSL with low IR emissivity, while microwave radar stealth is realized through absorption and transmission hybrid mechanisms under *y*‐ and *x*‐polarizations. Moreover, AMTS with polarization selection and polarization conversion functions mainly contributes to the desired forward communication/detection function based on precise curvature phase compensation when fed by *y*‐polarized horns from the bottom. Subsequently, we will provide a detailed description of the design of each layer.

### 2.1 Design and Characterization of IRSL

**Figure 2:**
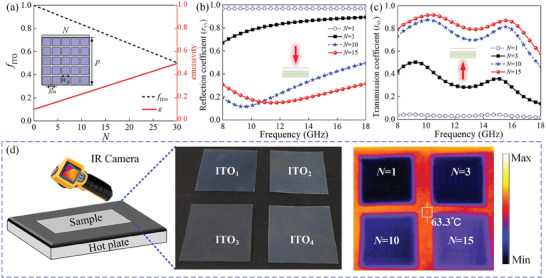
Characterization of meta‐atom with different ITO patch periods under microwave and IR wave detection. a) Theoretically calculated filling ratio of ITO (*f*_ITO_) and IR emissivity (*ε*) varying with ITO patch periods *N*. b) FDTD calculated backward reflection (*r*_yy_) and c) forward transmission coefficients (*t*_xy_) under *y*‐polarized microwave excitation with different *N*. Here, the subscriptions *x* and *y* indicate different polarizations. d) Schematic diagram of IR testing setup and thermal IR images of four fabricated samples with different ITO patterns: ITO_1_ (*N* = 1), ITO_2_ (*N* = 3), ITO_3_ (*N* = 10), and ITO_4_ (*N* = 15).

According to Stefan–Boltzmann's law *Q* = *εσT^4^*, the total emitted radiation *Q* of a target is determined by its surface emissivity *ε* and surface temperature *T*. *σ* represents the Stefan‐Boltzmann constant. In principle, IR radiation intensity of the target is higher than that of background environment, thus materials with low emissivity are essential for IR camouflage. Herein, ITO squared patch films etched on polyethylene terephthalate (PET) films are utilized as IRSL. Different from characteristics in microwave band, dielectric constants of ITO in IR band meet Drude model, and its specific parameters are presented in Figure S1 (Supporting Information). Simulation results indicate that ITO material with 6 Ω sq^−1^ exhibits metal‐like properties in IR band and maintains a low emissivity (*ε*_ITO_≈0.1) at 3–14 µm. However, such continuous ITO film with a low sheet resistance will cause strong microwave reflection, which severely contradicts microwave camouflage. Therefore, each ITO patch film with a period of *p* = 12 mm is divided into *N* square patches with a size of *w*_n_, and the gap between adjacent squares is *g*_n_ = 0.1 mm, see illustration in **Figure** **2**a. Theoretically, IR emissivity *ε* of whole meta‐atom can be calculated as follows.

(1) $$\varepsilon =\varepsilon_{ITO}f_{ITO}+\varepsilon_{PET}f_{PET}$$

where *f*_ITO_ and *f*_PET_ are the filling ratio of ITO and PET films, respectively, and *f_I_*_TO_ + *f*_PET_ = 1. *ε*_PET_ denotes the emissivity of PET substrate. Here, *ε*_ITO_ = 0.1, *ε*_PET_ = 0.9, *f*_ITO_ and the variation of emissivity *ε* with the number of patches is depicted in Figure 2a. It is obvious that *f*_ITO_ decreases linearly with the increase of *N* while *ε* increases gradually. To investigate the impact of *N* on microwave properties of asymmetric metasurface, EM‐wave simulations are conducted using commercial software CST. The finite‐difference time‐domain (FDTD) calculated reflection (*r*_yy_) and transmission (*t*_xy_) characteristics in 8–18 GHz are presented with different *N* in Figure 2b,c, respectively. All the results demonstrate that the reflectance of backward wave decreases and the transmittance of forward wave increases as *N* increases, thereby facilitating efficient transmission within wideband microwave stealth. The characterization of *x*‐polarized case is detailed in Figure S2 (Supporting Information), where efficient microwave stealth and transmittance is observed when *N* exceeds 10. By comprehensively taking both IR and microwave properties into consideration, *N* = 15 is ultimately chosen, whose transmission coefficient *t*_xy_ is basically above 0.8, and reflection coefficients remain below −10 dB across 8–16 GHz. To further verify IR characteristics, four different ITO patterns (*N* = 1, 3, 10, and 15) with the same size of 36 ×36 mm^2^ are tested using an IR thermal imager, as shown in Figure 2d. All ITO film samples were placed on a heating plate with a background of around 60 °C. As expected, the surface temperature of ITO samples increased with the increase of *N*, indicating that large *N* results in a deterioration of IR camouflage performance (increased IR emissivity).

### 2.2 Design and Analysis of MAL

**Figure 3:**
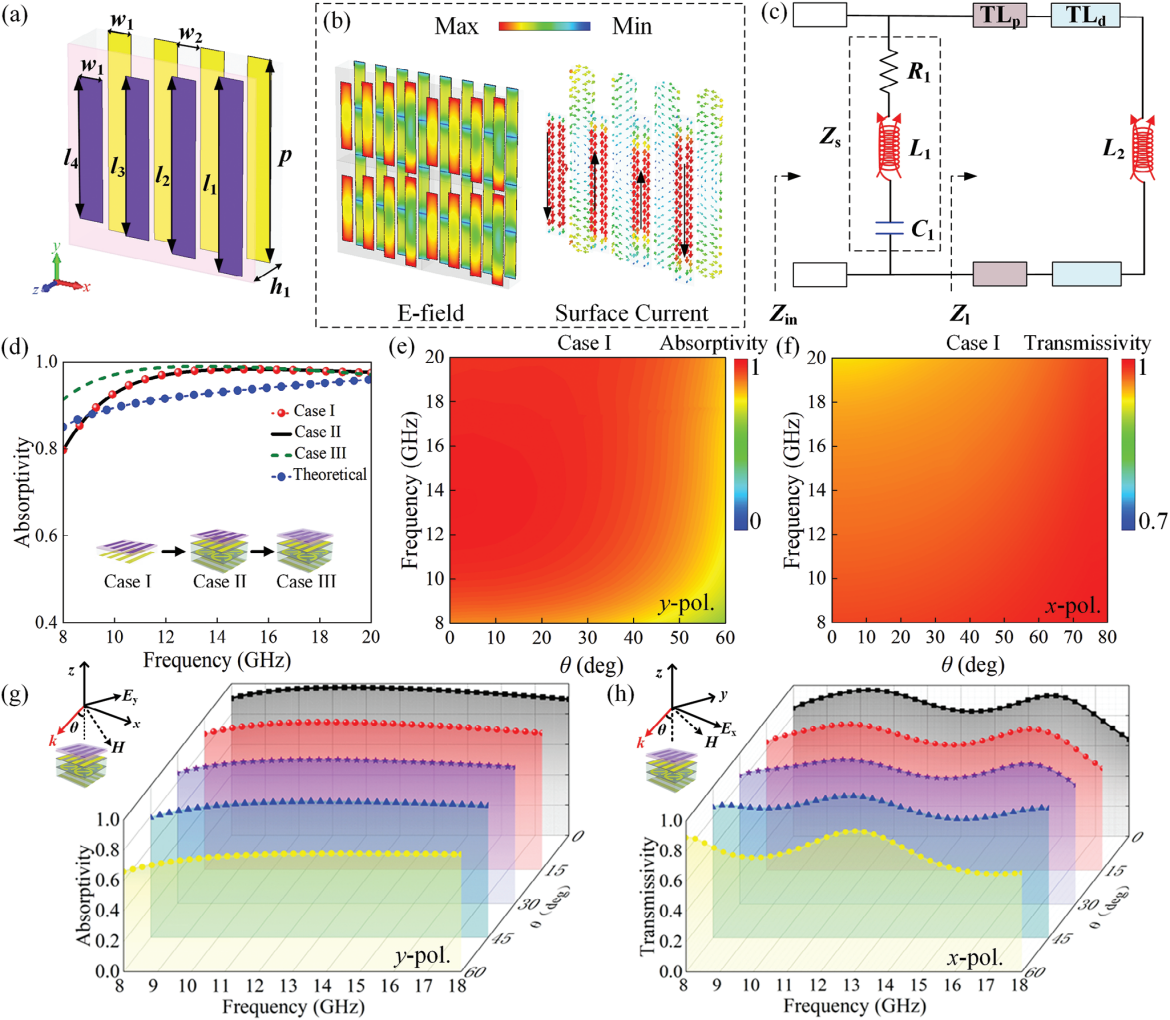
Theoretically calculated and numerically simulated full‐wave EM response of the sandwiched absorber composed of MAL. a) Topology of sandwiched absorber comprising MAL, PMI foam, and metal grating. The geometrical parameters are *p* = 12, *l*_1_ = 11.5, *l*_2_ = 10.5, *l*_3_ = 9.5 *l*_4_ = 8.5, *w*_1_ = 1.5, *w*_2_ = 1.5, and *h*_1_ = 3 mm. b) Distribution of E‐field and surface current at 14 GHz. c) Equivalent circuit model of the absorber with *R*_1_ = 200 Ω, *C*_1_ = 0.078 pF, *L*_1_ = 0.736 nH, and *L*_2_ = 0.883 nH. d) Theoretical and simulated absorptivity for three scenarios: Case I is the proposed sandwiched absorber, Case II is the integration of MAL and AMTS, and Case III represents the entire meta‐atom. Numerically simulated e,g) absorptivity and f,h) transmissivity of (e,f) the sandwiched absorber in Case I and (g,h) the entire meta‐atom in Case III under *y*‐ and *x*‐polarized wave incidence.

To engineer a light‐weight and low‐cost metadevice for microwave camouflage, a sandwiched absorber is formed by integrating MAL, polymethacrylimide (PMI) foam, and metal grating along *y*‐axis, as shown in **Figure** **3**a. Here, strip array patterned ITO films with a sheet resistance of 100 Ω sq^−1^ are employed on MAL, and the four strips with gradient lengths effectively broaden the absorption bandwidth by generating multiple resonances. PMI foam with a thickness of *h*_1_ and a dielectric constant *ε*_r_ = 1.07 (1‐j0.0026) is adopted for support due to easy conformation. To function as a ground for selectively absorbing *y*‐polarized waves, the width and arrangement of the metal gratings are designed to match those of the top ITO strips. Finally, the optimum geometric parameters are illustrated in the caption of Figure 3 according to the parametric study (see Figure S3a–c, Supporting Information). The physical mechanism of sandwiched absorber is further inspected from E‐field and current distributions at 14 GHz, as depicted in Figure 3b. Obviously, positive and negative charges accumulate at the respective ends of ITO strip film, thereby forming a capacitor. Meanwhile, the surface currents are evenly distributed along the strips, which can be considered as an inductor. Therefore, the equivalent circuit model of such absorber is presented in Figure 3c, where the MAL is modeled by a series circuit of resistance *R*_1_, capacitance *C*_1,_ and inductance *L*_1_, and finally the corresponding values are obtained as *L*_1_ = 0.736 nH, *L*_2_ = 0.883 nH, *R*_1_ = 200 Ω, and *C*_1_ = 0.078 pF according to empirical formula in.^[^ ^52^ ^]^ Moreover, the metal grating is modeled by inductance *L*_2_, PET, and PMI foam substrates are represented by transmission lines (TL_p_ and TL_d_), more details can be seen in Section S3 (Supporting Information), and the absorptivity can be obtained as

(2) $$A=1-{\left|\frac{Z_{in}-Z_{0}}{Z_{in}+Z_{0}}\right|}^{2}$$

where *Z_0_* = 377 Ω is the free‐space impedance, and Z_in_ denotes the input impedance, which can be calculated according to Equation (3)

(3) $$Z_{in}=\frac{Z_{l}Z_{s}}{Z_{l}+Z_{s}}$$

where Z_s_ and Z*_l_* represent the characteristic impedance of ITO film and load impedance, respectively. Furthermore, the comparison of simulated absorptivity in three cases and theoretically calculated one based on Equation (3) under *y*‐polarized wave incidence are portrayed in Figure 3d, demonstrating that the absorptivity remains above 0.8 within 8–20 GHz. Here, Case I is the sandwiched absorber, while Case II incorporates an additional underlying AMTS, Case III is the entire meta‐atom. Obviously, the absorptions of Cases I and II are essentially identical, while that of Case III exhibits a significant increase at lower frequencies, which can be attributed to the improved impedance matching facilitated by top IRSL. Moreover, the sandwiched absorber exhibits high absorptivity over 0.8 within 8–20 GHz from incidence angle of 0° to 60°, while achieves over 90% transmissivity within 0°∼80° for *x*‐polarized wave, see Figure 3e,f. Full‐polarized stealth is achieved at large angles through combination of absorption and transmission, as arbitrary polarized wave can be decomposed into orthogonal polarized states. More results under various polarizations are illustrated in Figure S3d (Supporting Information). In addition, angle insensitivity of the entire meta‐atom is further illustrated through simulations at various incident angles (0°– 60°, with an interval of 15°) in microwave band, as shown in Figure 3g,h. The independence of absorption behavior on the incident angle allows for achieving microwave stealth at large oblique angles up to 60°, regardless of the location of detectors.

### 2.3 Conformal Design of AMTS

**Figure 4:**
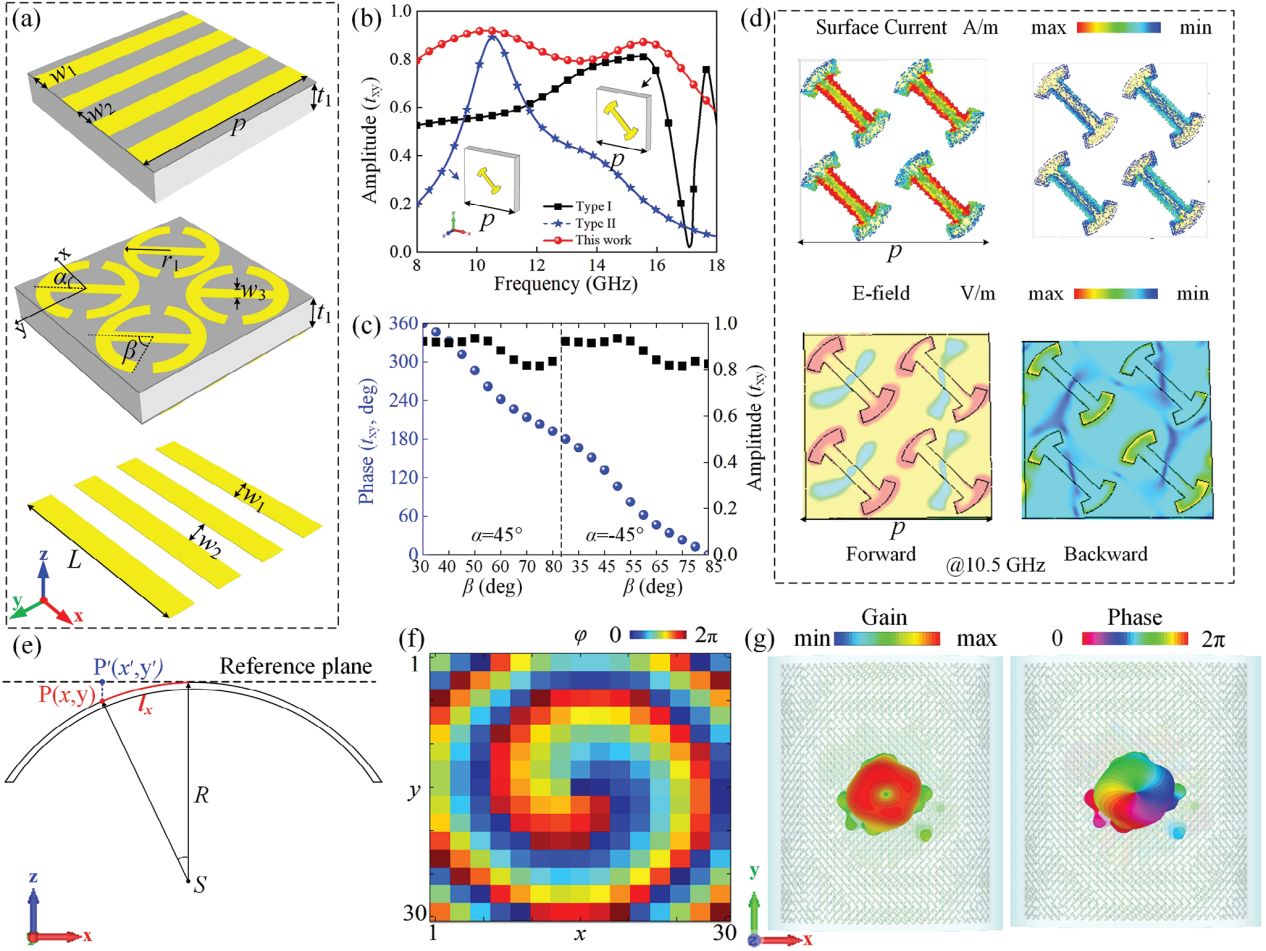
Design and numerical characterization of AMTS and final asymmetric composite metasurface in curved architecture. a) Topology of the AMTS meta‐atom. Two 3D printing ABS‐M30 boards (*ε*_r_ = 2.7, tan*δ* = 0.005, and *t*_1_ = 2 mm) are sandwiched by triple‐layer metallic patterns. The geometric dimensions of two orthogonal gratings are identical with *L* = *p* = 12 and *w*_1_ = *w*_2_ = 1.5 mm. To ensure high efficiency on a curved platform, four quasi‐I‐shape resonators also share the same parameters of *α*, *β, r*_1_ = 2.5, and *w*_3_ = 0.8 mm. b) Comparison of transmission amplitude among different types of I‐shaped structures, where single‐I shape with *r*_1_ = 2.5 mm for Type I and *r*_1_ = 5 mm for Type II while *r*_1_ = 2.5 mm in four‐I resonators. Other parameters remain unchanged (*β* = 30°, *α* = 45°). c) The amplitude and phase curves of *t*_xy_ versus *β* at *α* = ±45°. d) FDTD calculated surface current and electric‐field distributions under forward and backward incidence at 10.5 GHz. e) Diagram of curved phase compensation for metasurface with cylindrical platform based on ray‐tracing method. Point *S* represents the position of ideal feeding source. *P* is a point on the curved surface and *P*ʹ denotes its projection on reference plane. f) Distribution mapping of theoretical phase *φ* for curved metasurface. g) 3D far‐field radiation pattern of composite curved metasurface at 10.5 GHz.

The key to achieving forward detective wavefront control is to design a meta‐atom with high transmission efficiency. Here, AMTS meta‐atom is composed of two ABS‐M30 plates sandwiched by a pair of orthogonal metallic gratings and four quasi‐I‐shape resonators, see **Figure** **4**a. The top grating not only serves as the ground of absorber to fully reflect *y*‐polarized wave, but also allows *x*‐polarized wave to transmit through, while the bottom grating can only transmit forward and backward *y*‐polarized waves. The middle metallic layer is composed of four quasi‐I‐shape resonators oriented along *α* = 45°, which breaks the symmetry along *x* and *y* axes and converts most incident power to its cross‐polarized component. To demonstrate the advantages of such four‐I structure, Figure 4b compares the EM behavior between our four‐I resonators and common single‐I structure with different *r*_1_ under normal incidence. Obviously, the proposed meta‐atom exhibits broadband and high transmission characteristics. In addition, our combined meta‐atom can effectively maintain angle‐insensitivity due to its miniaturization, which is crucial for designing conformal metadevices where oblique incidence is a common case. More details can be referred to Figure S4 (Supporting Information). When *y*‐polarized microwave is normally incident from the bottom, the meta‐atom exhibits a broadband high cross‐polarization conversion (*t*_xy_ > 0.8) across 8–16 GHz (Figure S5, Supporting Information). Moreover, a continuous phase change of *φ*_xy_ is obtained by adjusting the arm length angle *β* from 30° to 85°, as shown in Figure 4c. To achieve a full 2π phase coverage, an additional 180° phase shift is introduced by changing *α* from 45° to −45°, without altering *r*_xy_ significantly. In addition, the full amplitude and phase spectrum at all scanned frequencies and *β* under both *x*‐ and *y*‐ polarized wave incidence are further afforded in Figure S5c (Supporting Information). To further investigate its physical mechanism, Figure 4d illustrates the simulated surface current and electric field distributions of four quasi‐I‐shape resonators under forward and backward *y*‐polarized wave at 10.5 GHz. Apparently, the middle bar and the edges of circular I‐shape resonate strongly under illumination of forward *y*‐polarized wave. However, the energy is significantly attenuated when EM wave is incident from backward direction, indicating inherent asymmetric properties.

To accommodate arbitrary‐shaped targets in practical applications, a precise phase compensation based on ray‐tracing method is employed for a curved architecture. As shown in Figure 4e, the point *P* (*x*, *y*) on the designed curved metasurface is correspondingly mapped to *P*ʹ (*x*ʹ, *y*ʹ) on the reference plane when microwave is emitted from feeding source *S*. According to geometric relationship, *x*ʹ = *R*sin(*x*/*R*) and *y*ʹ = *y* should be satisfied on a cylindrical platform with a radius of *R*. By applying ray‐tracing method, the required compensated phase *δ* between SP and SPʹ can be calculated as

(4) $$\delta =\frac{2\pi }{\lambda }\left[R\left(1-\cos\frac{x}{R}\right)+\sqrt{R^{2}+y^{2}}-R\right]$$

In recent decades, vortex beams carrying OAM have garnered significant attention in radar communication/detection due to their capacity to obtain higher resolution combining both static and motion information.^[^ ^53^ ^]^ In addition, vortex beams exhibit extremely low RCS due to the phase singularity. Herein, spiral vortex beam generation in conformal platform is utilized as an illustrative example, the theoretical phase distribution after introducing vortex phase can be calculated as follows.

(5) $$\varphi =\frac{2\pi }{\lambda }\left[R\left(1-\cos\frac{x}{R}\right)+\sqrt{R^{2}+y^{2}}-R+l\arctan\left(\frac{y^{\prime }}{x^{\prime }}\right)\right]$$

where *l* is the topological charge of the OAM mode. For verification, a theoretical phase diagram with *l* = 1 and *R* = 102 mm is depicted in Figure 4f, and more details can be referred to Figure S6 (Supporting Information). Figure 4g further affords 3D far‐field radiation patterns of the proposed metasurface at 10.5 GHz when fed by a y‐polarized horn from the bottom, where the *x*‐polarized vortex‐beam with a high gain of 14.5 dB is achieved. In addition, the curved metasurface exhibits broadband performance within 8–18 GHz, see Figures S7 and S8 (Supporting Information). It should be noted that conformal scenario under microwave detection can be regarded as special case of planar architecture at oblique incidence. Therefore, the backward stealth performance will not be affected due to the angle‐insensitive sandwiched absorber.

## 3 Fabrication and Experiments

**Figure 5:**
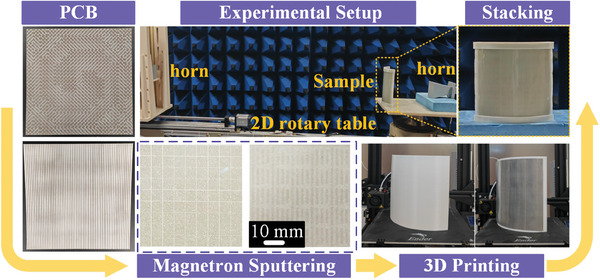
Illustration of the fabrication process and far‐field radiation experimental setup for the final asymmetric composite metasurface sample. All metallic patterns were printed on flexible F4B substrates by PCB technology, ITO films were fabricated via magnetron sputtering technology, and the support structures were manufactured using 3D printing technology to create a robust conformal framework for final stacking.

To verify the feasibility of the proposed strategy, a prototype was fabricated, assembled, and fixed by 3D printing external frame. As shown in **Figure** **5**, the sample was prepared based on a fabrication process by combining printed circuit board (PCB), magnetron sputtering, and 3D‐printing technique. Here, all metallic patterns were printed on flexible F4B substrates with a thickness of *h* = 0.1 mm, *ε*_r_ = 2.65, and tan*δ* = 0.001 to facilitate subsequent conformal assembly. ITO films with sheet resistances of 6 and 100 Ω sq^−1^ were deposited on 175‐µm‐thick PET substrates with *ε*_r_ = 3(1‐j0.06) via magnetron sputtering technology (see magnified view of MAL and IRSL depicted in the bottom panel of Figure 5). Conformal platform and external frame were prepared by using 3D printing technology. The hybrid metadevice is composed of *m*×*m* = 15 × 15 meta‐atoms, corresponding to an area of 180 × 180 mm^2^. The far‐field radiation patterns of the sample were measured using the setup illustrated in top panel and detailed microwave experiments in the Experimental Section.

**Figure 6:**
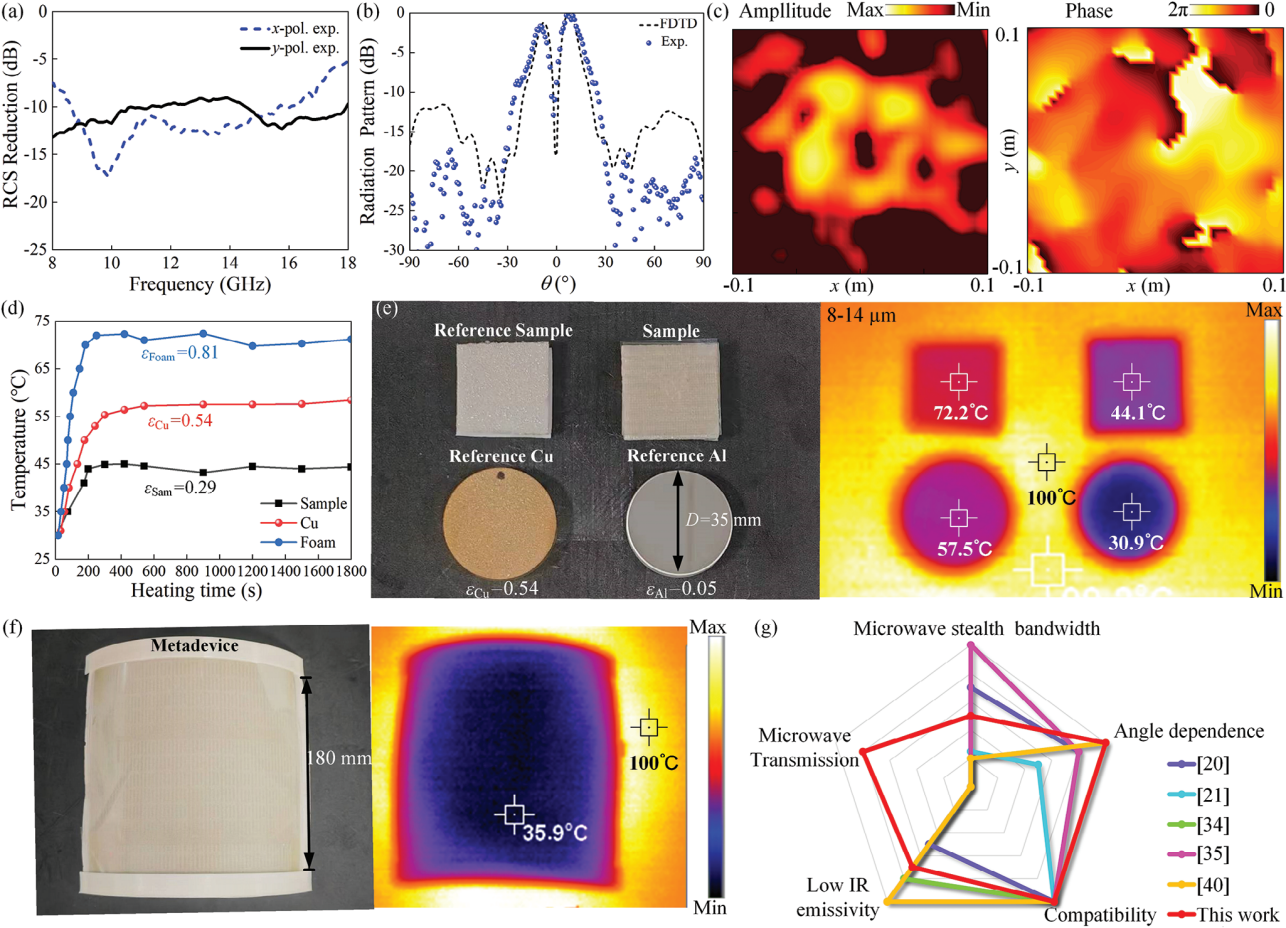
Experimental characterization of the fabricated asymmetric composite metasurface sample in microwave and IR bands. a) RCS reduction performance under backward detection of *x*‐ and *y*‐polarized waves. b) Normalized far‐field radiation patterns of forward detective vortex beam within −90°–90° in steps of 1°. c) Near‐field intensity and phase maps measured at 2D cross‐section aperture perpendicular to the detective vortex beam in transmission region. d) Apparent temperature variation over time of the fabricated metadevice and reference samples under ambient temperature heating from 27 to 100 °C. e) Optical photographs (left) and steady‐state IR thermal images (right) of composite metasurface and reference samples and f) metadevice at 8–14 µm. g) Comparison of compatible camouflage performance between this work and other material systems.

For verifications, we first characterize the microwave stealth performance when detected by both normally incident *x*‐ and *y*‐polarized waves, as depicted in **Figure** **6**a. The measured stealth bandwidth for 10 dB RCS reduction ranges from 8 to 18 GHz, corresponding to a fractional bandwidth of 76.9% for both two cases. Moreover, the composite metasurface is capable of maintaining a wideband RCS reduction under any polarization as EM wave can be decomposed into two orthogonal polarized states. To further verify the detective wavefront control, far‐field and near‐field experiments were performed at 10.5 GHz, as portrayed in Figure 6b,c. Distinct doughnut patterns with an amplitude null in center is observed, and the measured phase distribution manifests an obvious spiral profile varying 2π, which is an intrinsic characteristic of vortex beam with mode of *l* = 1. Compared with FDTD calculation, slight deviations in experiment may be partially to tolerances inherent in fabrication and assembly where multilayers are not exactly aligned with accurate spacer.

As to IR stealth, average IR emissivity of proposed compatible camouflage metadevice was measured by using an IR camera, as depicted in Figure 6d. Notably, our proposed metadevice exhibits outstanding thermal camouflage capability, maintaining a surface radiation temperature below 45 °C for 30 min on the 100 °C heating stage. However, the temperature of the foam sample without top IRSL exceeds 70 °C, whereas that of reference copper is maintained at 55 °C. This is mainly because the ITO structure with high filling ratio on IRSL provides low emissivity to realize IR stealth. More details for comparison can be referred to Figure S9 (Supporting Information). To characterize emissivity, we conducted a comparative analysis with Cu (*ε*_Cu_ = 0.54) and Al (*ε*_Al_ = 0.05) standard reference samples, as depicted in Figure 6e,. Here, all samples were compared at a stable ambient temperature of 100 °C after heating for 5 min. By comparing apparent temperatures with that of standard reference sample (Cu and Al), the emissivity at 8–14 µm can be approximately calculated as *ε*_Foam_ = 0.81, *ε*_sample_ = 0.29, and *ε*_metadevice_ = 0.14. In addition, IR‐2 dual band emissivity meter is utilized to further evaluate the IR stealth property, see Figure S10 (Supporting Information). Measurement results indicate that the proposed metadevice exhibits an emissivity of 0.283 within 8–14 µm and 0.186 within 3–5 µm, demonstrating elegant IR stealth characteristics. To the best of our knowledge, such a comprehensive camouflage system has not been previously reported, and the comparison of multispectral compatible camouflage performances between this work and prior work are shown in Figure 6g. Our strategy not only enables efficient modulation of microwave transmissive wavefront but also maintains excellent IR‐microwave compatible camouflage. Lastly, our proposed metadevice is capable of inherent mass‐production at a low cost.

## 4 Conclusion

To sum up, we have proposed and experimentally demonstrated a new paradigm of asymmetric composite metasurface integrated detection and anti‐detection with microwave‐IR compatible camouflage. IR stealth is realized by high filling ratio of ITO patches on the IRSL, microwave stealth is achieved based on absorption under *y*‐polarization while transmission for *x*‐polarization. The analysis of the sandwiched absorber composed of MAL and AMTS were conducted by equivalent circuit model and EM field distributions. Moreover, microwave detective wavefront control is obtained by precise phase compensation on AMTS in curved architecture based on ray‐tracing method. For verification, a metadevice is fabricated and tested in both microwave and IR bands. Experimental results have verified all predesigned functions with high efficiency, showcasing forward spiral vortex‐beam generation for communication/detection, as well as wideband backward RCS reduction and low IR emission for anti‐detection. With the combination of multispectral stealth mechanism and low‐cost fabrication techniques on a curved platform, our strategy opens up a new way to achieve detective wavefront control while radar‐IR compatible stealth, which exhibits promising applications in modern detection, defense, and multispectral stealth system.

## 5 Experimental Section

### Numerical Characterizations

All numerical designs and FDTD characterizations were performed through simulation package CST Microwave Studio. In calculations of meta‐atom, periodic boundary conditions were adopted to mimic infinite plate. It was worth noting that settings of ITO were different in microwave and IR bands. The Drude model was used in IR band, while sheet resistance mode was employed for microwave characterization. To realize conformal structure model, the pre‐designed planar structure was bent onto the cylinder surface. In calculating radiation characteristics of detective OAM‐beam generator, open condition was set to the ends of inhomogeneous array.

### IR Measurement

The thermal IR images were captured using a thermal imaging camera (Smart Sensor ST9450) that operates within wavelength range of 8–14 µm. Two standard circle plates with a diameter of 35 mm were utilized here as reference. One was an aluminized benchmark plate with a low emissivity of 0.05, and the other was a copper reference plate with a medium emissivity of 0.54. The measured sample must be flat. To minimize measurement error, sample pieces with 36 × 36 mm^2^ were randomly cut from the as‐prepared sample to investigate IR camouflage performance. IR emissivity was further measured by an IR‐2 dual band emissivity meter, which operates within 3–5 and 8–14 µm. More detailed characterization methods are provided in Note S6 (Supporting Information).

### Microwave Experiments

The far‐field experiment was carried out using an automatically moved 2D rotary table, see Figure 5. A pair of broadband linearly polarized horns within 2–18 GHz were utilized as transmitter and receiver. During the experiments, the transmitted horn was fixed while the received horn was mounted on an automatically rotating arm, which was controlled by an electronic motor in a circumference at a step of 1°. In far‐field experiments, the data were averaged ones after several repetitive measurements to eliminate random errors induced by the environment. For near‐field measurement, the probe was fixed to a 2D electronic step motor that could move automatically in an area of 0.1 m × 0.1 m with a step resolution of 10 mm. As to the experimental setup for reflection case, two linearly polarized horn antennas functioning as the receiver and transmitter were placed on the same side in front of the sample, and they were connected to an AV3672B vector network analyzer to record the static EM signals.

## Conflict of Interest

The authors declare no conflict of interest.

## Supporting information



Supporting Information

## Data Availability

The data that support the findings of this study are available in the supplementary material of this article.
