# Supplementary material for: Detection and Anti‐Detection with Microwave‐Infrared Compatible Camouflage Using Asymmetric Composite Metasurface
Source: Adv Sci (Weinh). 2024 Sep 24;11(43):2410364. doi: 10.1002/advs.202410364 (PMC11578305; doi:10.1002/advs.202410364)
Supplement: Supplementary file 1 — Supporting Information [file ADVS-11-2410364-s001.docx]

Supporting Information

Detection and Anti-detection with Microwave-Infrared Compatible Camouflage Using Asymmetric Composite Metasurface

Yanzhao Wang†, Huiling Luo†, Yanzhang Shao, Hui Wang, Tong Liu, Zhengjie Wang, Kai-yue Liu, Xiaogang Su, He-Xiu Xu*

1. **Permittivity and numerical characterization of ITO in IR band**

The permittivity and numerical characterization of ITO calculated by Drude model are given in **Figure S1**, here EM-wave simulations are conducted using CST Microwave Studio, applying the Drude model for ITO (epsilon infinity *ε*_b_=3.95, plasma frequency *ω*_p_= 3.07 × 10^15^ s^−1^; damping constant of *ω*_c_ = 1.82 × 10^14^ s^−1^). Besides, periodic boundary conditions are set along both *x*- and *y*-directions in FDTD simulation. As shown in Figure S1a, a negative dielectric constant indicates that ITO exhibits metal-like properties in IR band. According to the thermal radiation relation, the emissivity *ε* is equivalent to absorption rate, which can be calculated as *ε*=1-*R*-*T*, where *R* and *T* represent reﬂectivity and transmissivity, respectively. The simulated results indicate that the average emissivity of ITO is about 0.1 at 3~14 μm, see Figure S1b.


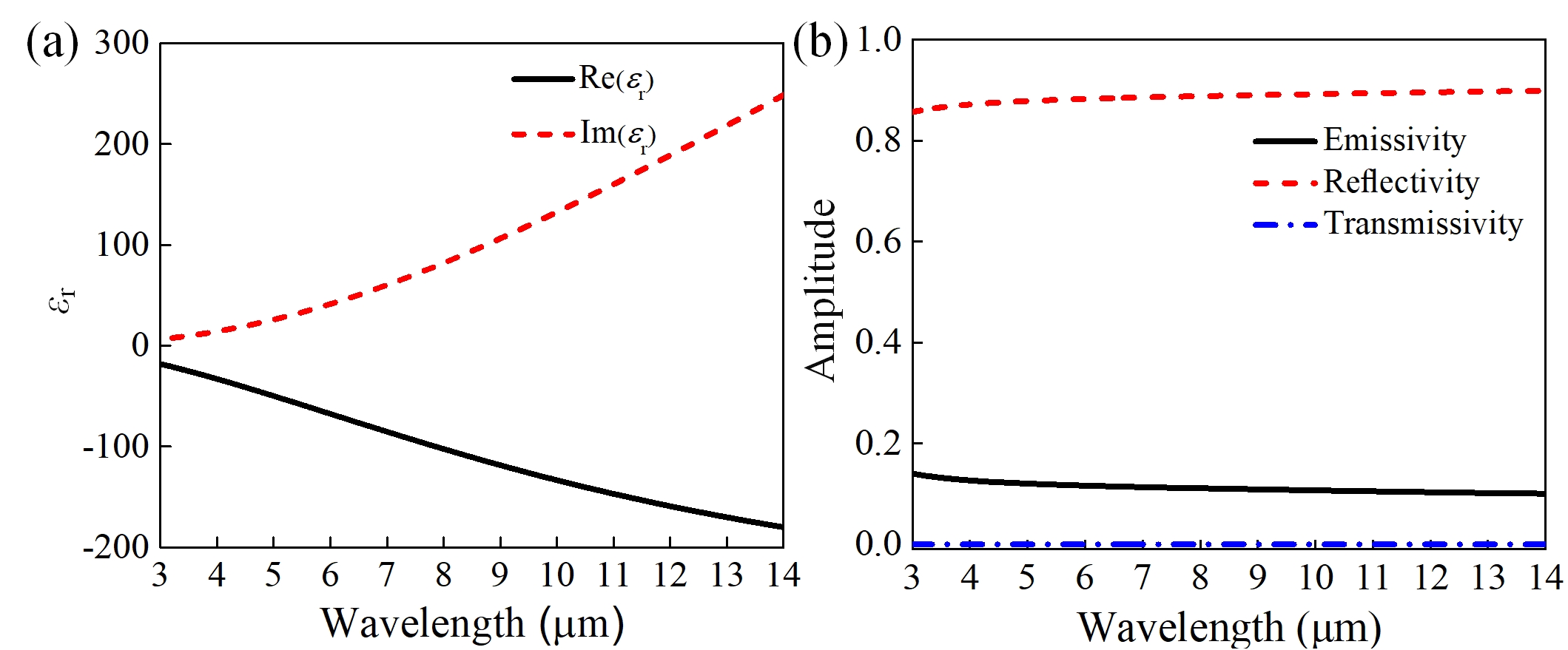


**Figure S1.** Characteristics of ITO films in IR band. (a) Permittivity of ITO across 3–14 μm. (b) Simulated emissivity, transmissivity, and reﬂectivity of ITO film.

1. **Additional results of meta-atom in microwave band**

In the main text, Figure 2b illustrates the reflection coefficient (*r*_yy_), demonstrating good microwave stealth in *y*-polarization case. To further investigate the microwave stealth effect under *x*-polarization, **Figure S2** portrays the reflection and transmission coefficients of meta-atom with different *N*. It is evident that an increase in *N* exhibits a significant impact on the reflection coefficient in a wide band. Moreover, for the transmission coefficient, the transmission efficiency increases with the increase of *N*, and efficient transmission stealth under *x*-polarization is observed when *N* exceeds 10. Considering the IR and microwave characteristics comprehensively, *N*=15 is selected. The numerical results show that *t*_yx_ is basically above 0.8 across 8~16 GHz, and *r*_xx_ is around under 0.4 for microwave stealth.


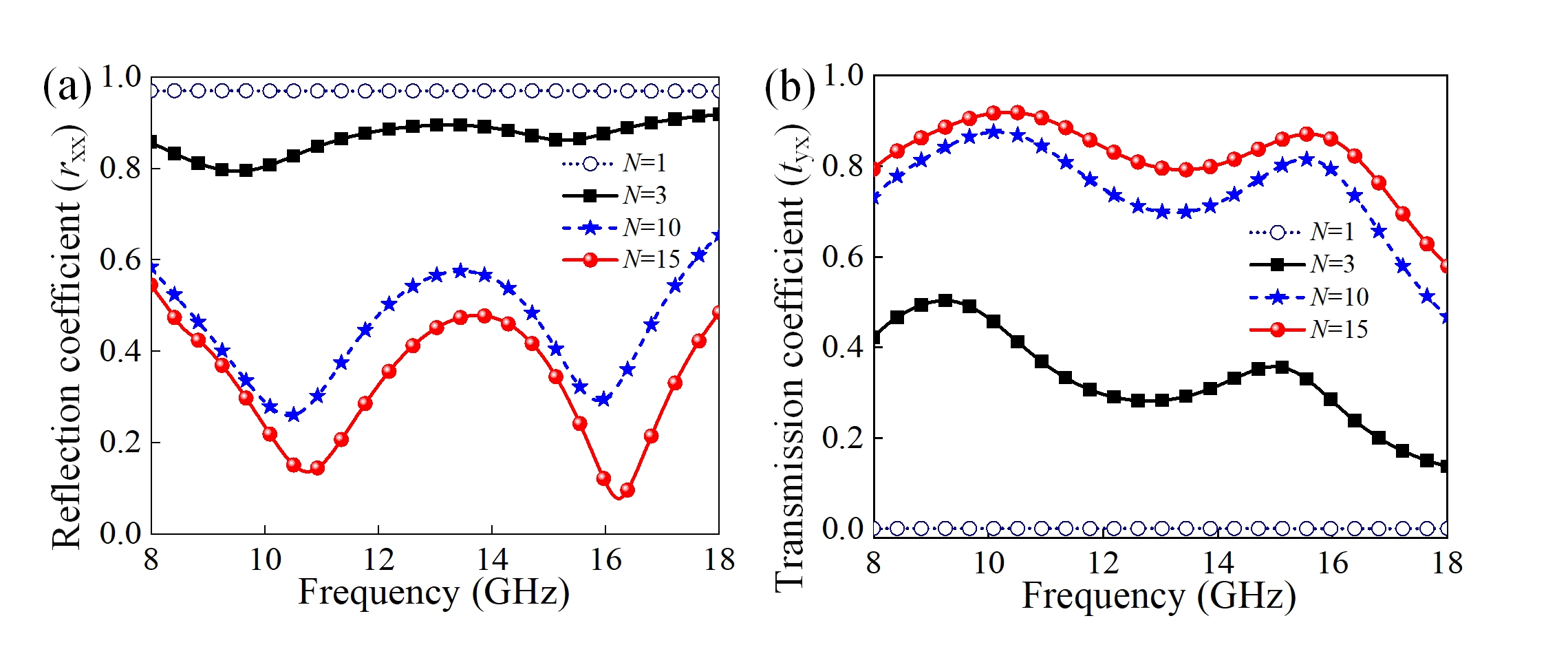


**Figure S2.** Characterization of microwave radar stealth. (a) Reflection coefficient (*r*_xx_) and (b) transmission coefficient (*t*_yx_) under *x*-polarized wave excitation along -z-axis with different *N*.

In general, the bandwidth of ITO-based absorber is primarily determined by the thickness and surface resistance of ITO film. To further afford guidance of the optimum design, a parametric study is carried out, as shown in Figure S3 a-c. It is obvious that the absorber with 100 Ω/sq^-1^ ensures high absorptivity across a broad frequency range of 8~18 GHz. The absorption peaks gradual decrease with a slight red shift as *h*_1_ increase from 2 to 3.5 mm when other parameters remain unchanged, see Figure S3c. Considering both bandwidth and reflection coefficient comprehensively, a thickness of *h*_1_=3 mm is ultimately selected. To demonstrate the feasibility of our absorber under different polarizations, Figure S3d presents EM spectrum of MAL excited at various polarization angles. Most importantly, the reflection coefficient consistently remains below -10 dB within 6~22 GHz when polarization angle *Φ* varies from 30° to 90°. Interestingly, a larger polarization angle result in a better performance especially when *Φ*=90°, where microwaves completely transmit through the designed MAL under *x*-polarized wave incidence.


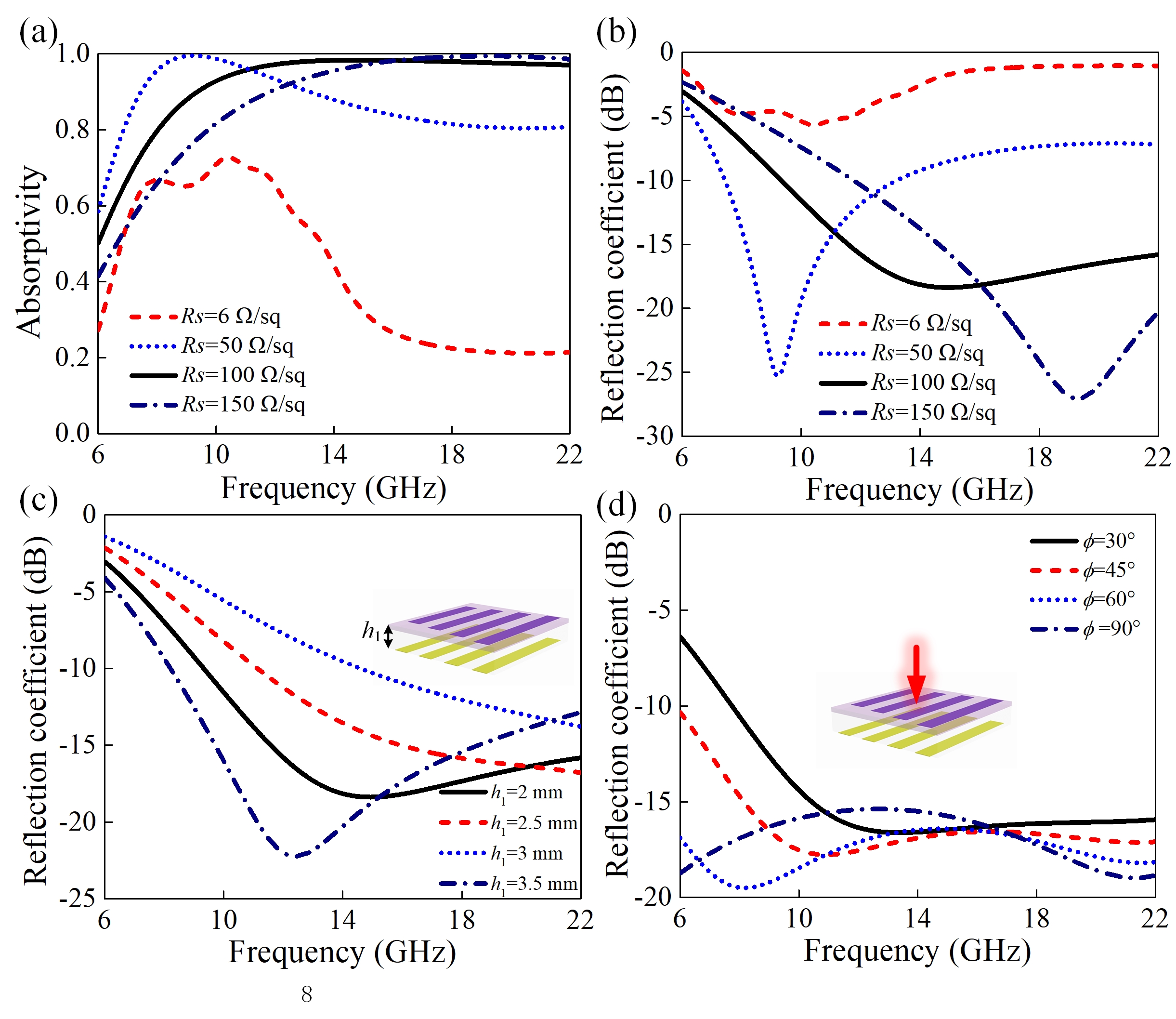


**Figure S3** Characterization of the proposed sandwiched absorber consisting of MAL. Numerical (a) absorptivity of sandwiched absorber with different *R*s and (b-d) reﬂection coefﬁcients with different (b) *R*s, (c) thickness *h*_1_, and (d) polarization angle *Φ*.

In the main text, Figure 4b compares our meta-atom with common single-I structure under normal incidence, Figure S4 further illustrates the comparison at an incident angle of *θ*=30°. It can be seen that our meta-atom with four quasi-I-shape resonators remains stable properties under oblique incidence, maintaining high transmission and wideband RCS reduction across 8-18 GHz. However, the results of the other two types of meta-atoms change significantly at *θ*=30°. When designing a conformal metasurface, it is better to keep a constant amplitude of meta-atom before and after conformation. In terms of meta-atom, the performance under conformal scenario is equivalent to that under specific oblique incidence. Therefore, meta-atom with angle-insensitivity is the best choice to adapt to different curvature structures. Additionally, combining multiple identical elements can also help mitigate the impact of conformation.

**
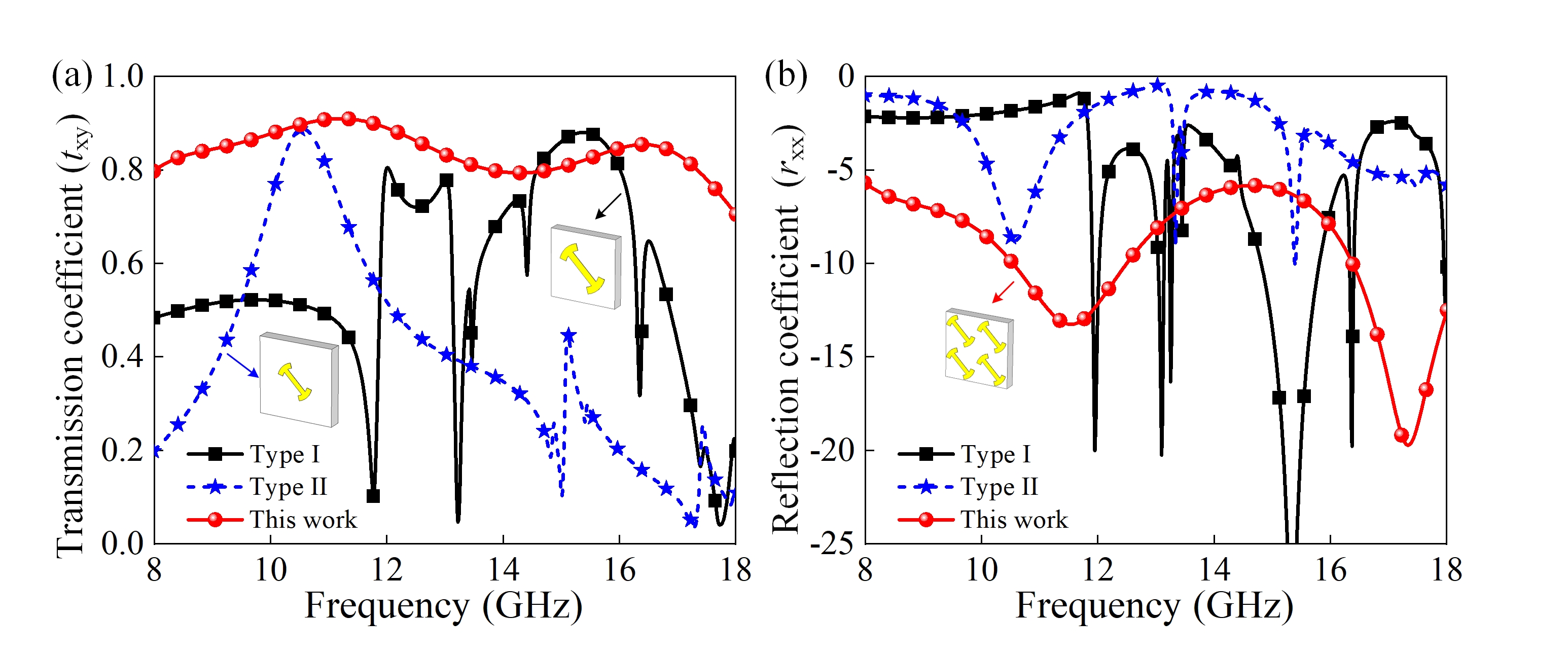
**

**Figure S4** Comparison of (a) transmission and (b) reflection coefficient of meta-atom with different types of I-shaped structures at *θ*=30°.

Characteristics of the whole meta-atom are obtained by performing full-wave numerical simulation before conformal design. Figure S5a confirms that the transmission rate of *t*_xy_ is higher than 0.9 over a broad band. Figure S5b affords the full amplitude and phase spectrum at all scanned frequencies and *β* under *y*-polarized wave incidence at *α*=45°. The meta-atom exhibits a broadband high cross-polarization conversion (*t*_xy_ > 0.8) across 8~16 GHz, and a phase difference of 180° can be achieved when *β* changes from 30° to 85° at *α*=45°. To further investigate the impact of changes in *α* and *β* on the microwave stealth performance, the reflection coefficients under both *x*- and *y*-polarizations are presented in Figure S5c and S5d. Obviously, microwave performances under *y*-polarization are basically unaffected by variations in *β* and *α*, maintaining -10dB within 8~18 GHz. In contrast, although *x*-polarized waves are affected by changes in *α*, the overall level is basically below 0.4, which is equivalent to -8 dB.


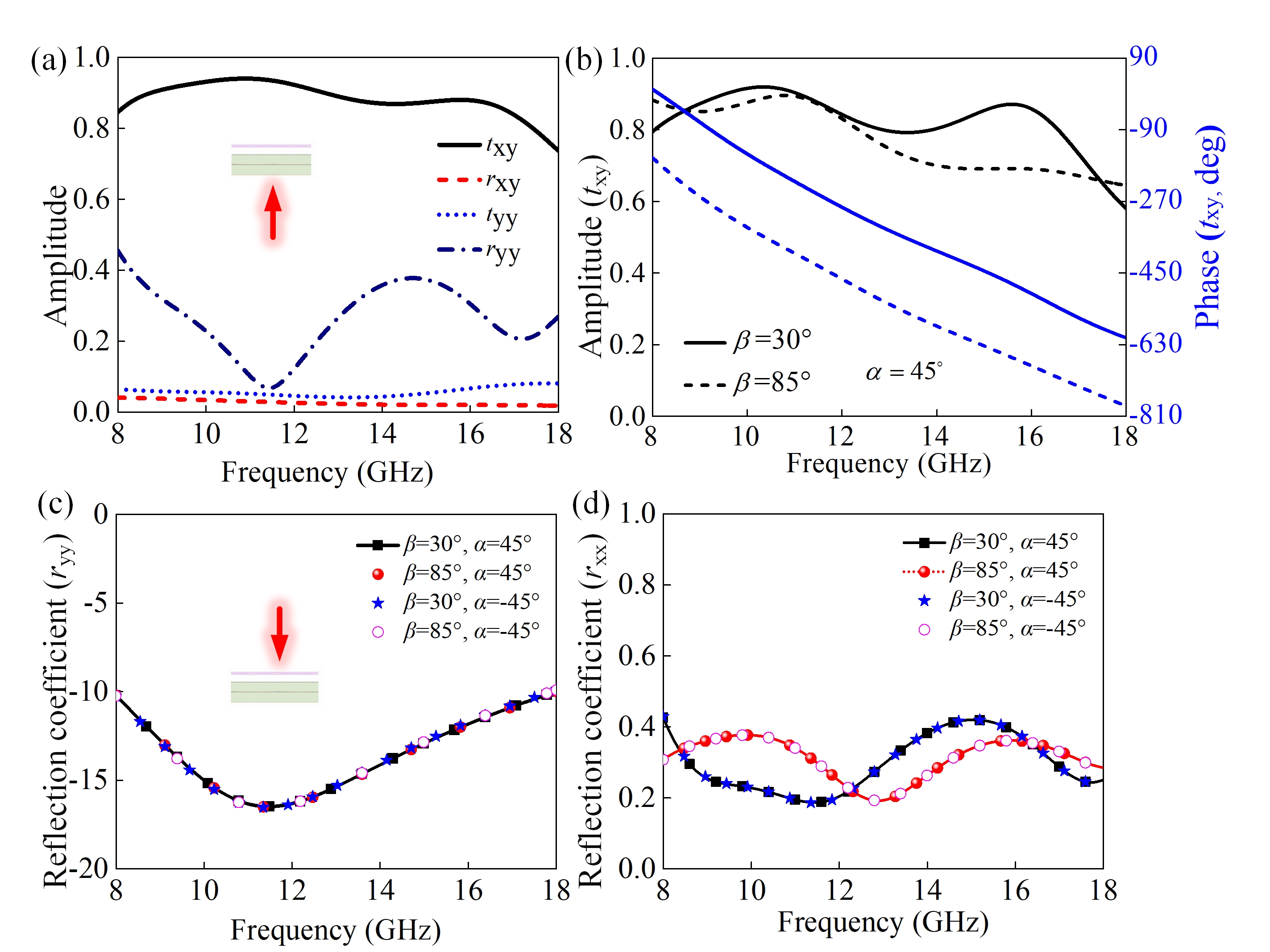


**Figure S5** FDTD calculated reflection and transmission coefficients of the meta-atom. (a) Reflection (*r*_xy_, *r*_yy_) and transmission (*t*_xy_, *t*_yy_) spectrum under *y*-polarized wave incidence from the bottom. (b) Transmission amplitude and phase of the meta-atom with *α*=45°. Reflection spectrum with different *β* and *α* under (c) *y*- and (d) *x*-polarized backward wave excitation.

1. **Equivalent circuit model analysis**

According to section 2.2, ITO ﬁlm was modeled by an *R*–*L*–*C* series circuit with the characteristic impedance Zs, which can be calculated as

$Z_{s}=R_{1}+j\omega L_{1}+\frac{1}{j\omega C_{1}}$ (S1)

The equivalent resistance *R*_1_ was calculated as *R*_1_=*R*s⋅S_unit_/S_eff_, where *S*_unit_ and *S*_eff_ represent physical area of the entire meta-atom and effective area of the patch pattern, respectively. The metal grating is equivalent to inductance *L*_2_, thus its impedance is Z_1_=jω*L*_2_. According to transmission line theory, PMI foam and PET ﬁlm could be considered as transmission lines with speciﬁc impedance Z_d_ and Z_l_, respectively ^[42]^, where *β* denotes the propagation constant.

$Z_{d}=Z_{0}\frac{Z_{1}+jZ_{0}\tan(\beta_{1}h_{1})}{Z_{0}+jZ_{1}\tan(\beta_{1}h_{1})}$ (S2)

$Z_{l}=Z_{0}\frac{Z_{\text{d}}+jZ_{0}\tan(\beta_{2}t_{PET})}{Z_{0}+jZ_{\text{d}}\tan(\beta_{2}t_{PET})}$ (S3)

1. **Illustration of detailed design of conformal AMTS**

The optimal values of *α* and *β* for vortex beam with topological charge of *l*=1 can be calculated according to Figure 4f, whose diagrams are shown in Figure S6a, b. The arranged units are carried out and simulated with *R*=102 mm. On the basis of amplitude and phase profiles, the final layout of the middle metallic layer of the metasurface can be synthesized, see Figure S6c.


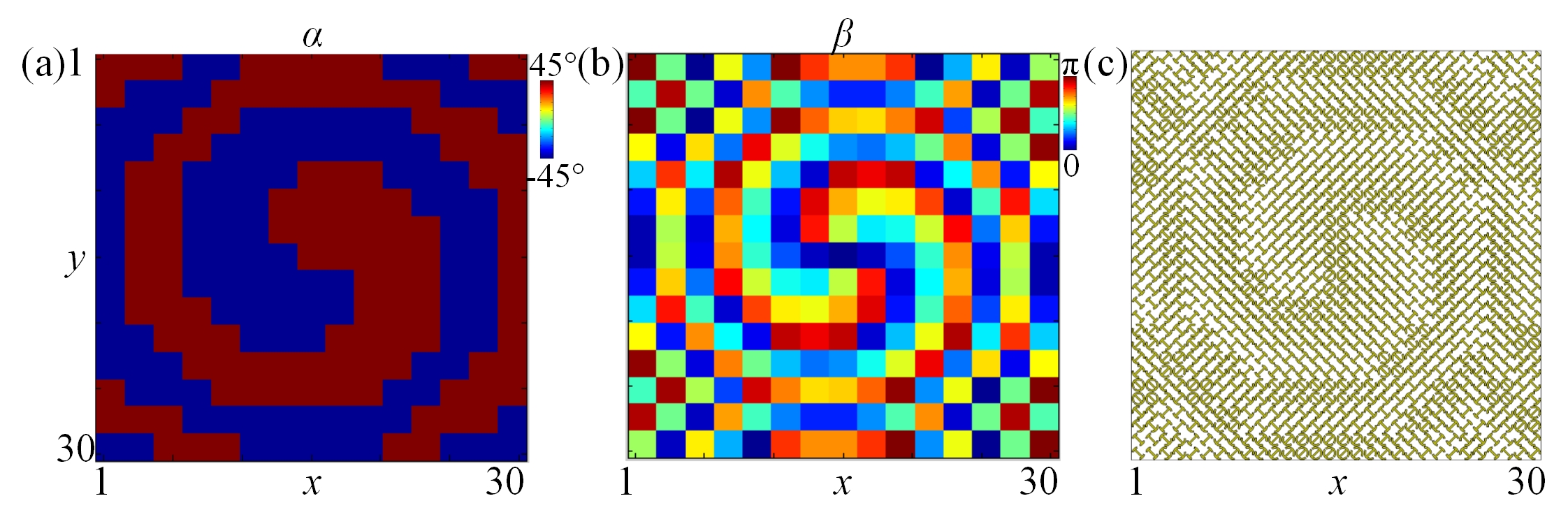


**Figure S6** Parametric illustration of the proposed metasurface. (a) *α*, (b) *β*, and (c) layout of the middle metallic layer.

1. **Additional results of vortex beam in microwave band**

In the main text, Figure 4 have illustrated the 3D far-field radiation patterns of the proposed curved metasurface at 10.5 GHz. Here, we afford more results to illustrate the broadband effect of the vortex beam, as shown in Figure S7 and S8. To verify the broadband characteristic of detection, 3D far-field radiation patterns at several typical frequencies are presented in Figure S7, where significant vortex beams are observed from 8 to 16 GHz. However, there is an evident degradation in the high-frequency pattern, and the symmetry of the hollow cone also deteriorates. To investigate the frequency-dependent variation of the vortex beam, simulated and measured radiation intensity maps from 8 to 18 GHz, covering a range of −90~90°, see Figure S8. Obviously, the experimental results of the main beam agree well with the simulated ones. However, the poor symmetry of the vortex beam and notably high sidelobes in the test are mainly due to conformal processing errors.


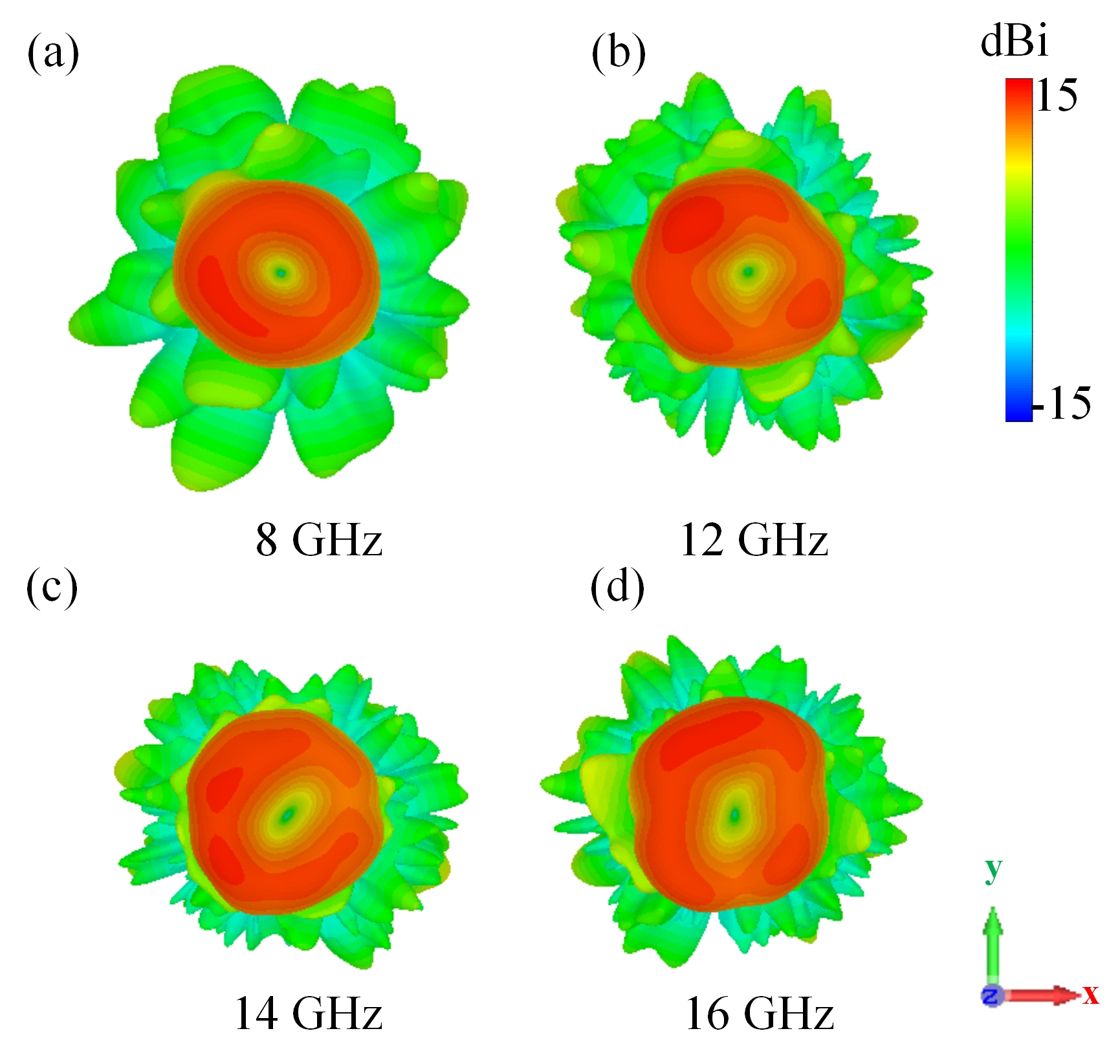


**Figure S7** Characterization of wide-band performance on vortex beam. FDTD calculated *x*-polarized 3D far-field radiation patterns at (a) 8 GHz, (b) 12 GHz, (c) 14 GHz, and (d) 16 GHz.


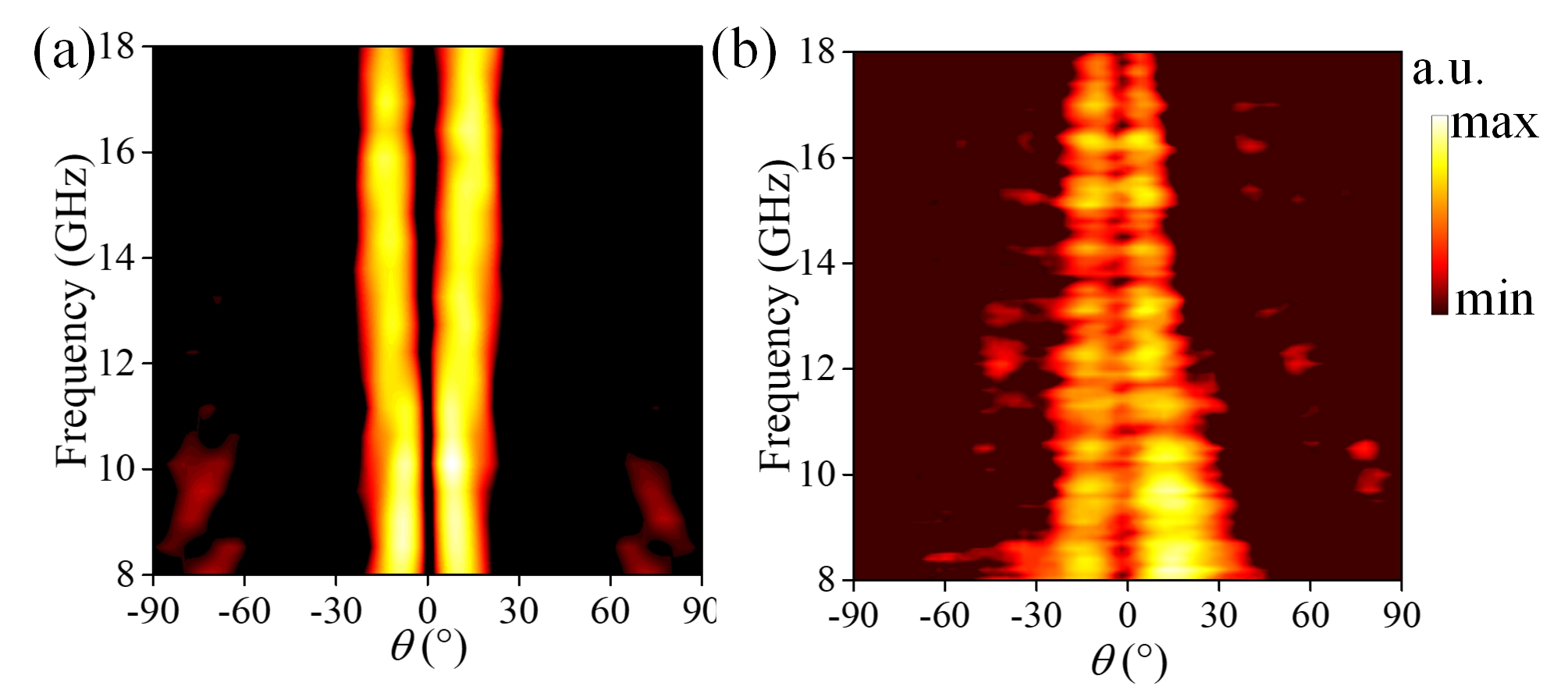


**Figure S8** (a) Numerical and (b) experimental characterization of the curved metasurface. All patterns are normalized to their maximum.

1. **Experimental characterization of low emissivity in IR dual bands**

To assess the adaptability of the designed metadevice to varying ambient temperatures, a heating plate is employed for temperature modulation. When the target temperature is set to 100℃, the variation curve of plate temperature is depicted in Figure S9a. In the main text, Figure 6 have illustrated apparent temperature variation and IR thermal images of the proposed metadevice and reference samples at 100 ℃. Furthermore, the apparent temperatures of the metadevice and reference samples were compared under two distinct ambient temperatures, as illustrated in Figure S9b, c. Obviously, lower apparent temperature indicates a better IR stealth performance. According to the apparent temperature of Cu and Al with known emissivity of *ε*_Cu_=0.54 and *ε*_Al_=0.05, it can be calculated that IR emissivities of the fabricated metadevice sample are 0.24 and 0.28 at ambient temperature of 27.3℃ and 34.9℃, respectively, indicating an increase in emissivity with rising temperature.


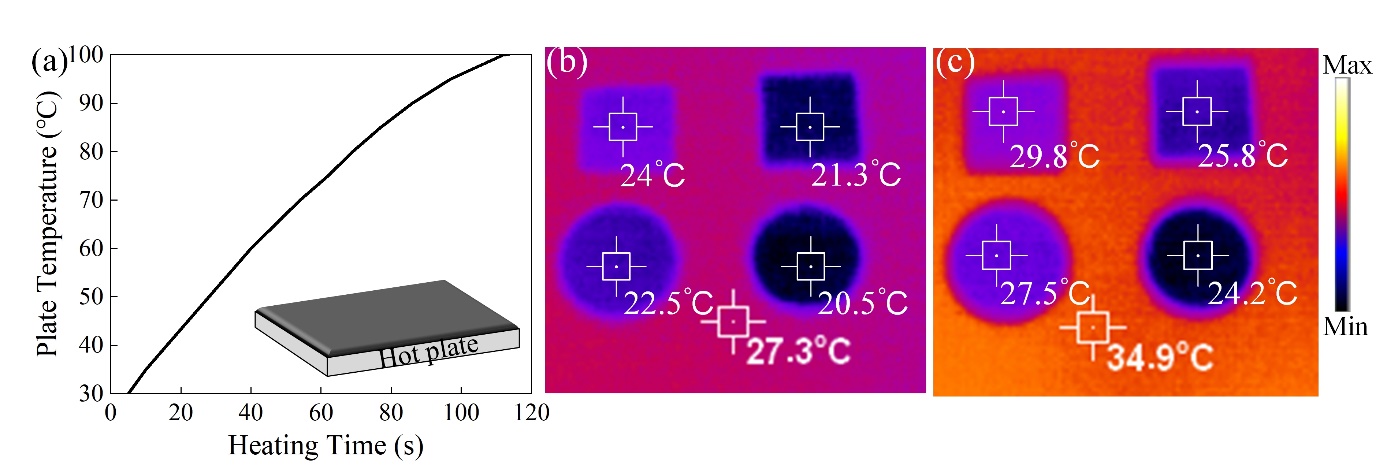


**Figure S9** (a) Plate temperature variation. IR thermal image (steady-state) of different samples under (b) 27.3 ℃ and (c)34.9 ℃ during thermal measurements in a practical environment.

To further investigate the IR stealth characteristics across different spectral bands, experiments were conducted within both 8-14 μm and 3-5 μm by an IR-2 dual band emissivity meter, as illustrated in Figure S10a, b. The average emissivity values were measured to be 0.283 and 0.186 at 8-14 μm and 3-5 μm, respectively. Both indicate low IR emissivity, which satisfy the requirements of IR stealth. Figure S10c further presents experimental measurements of emissivity in 3-5 μm range at a high ambient temperature of 100°C. During the heating test, the temperature control device is set to a specific temperature, and the emissivity can be measured once the heating furnace stabilizes at the required temperature. Despite the impact of temperature change on stealth capability, it is worth noting that even with an increase in emissivity to 0.315, the proposed metadevice still exhibits good IR camouflage performance.


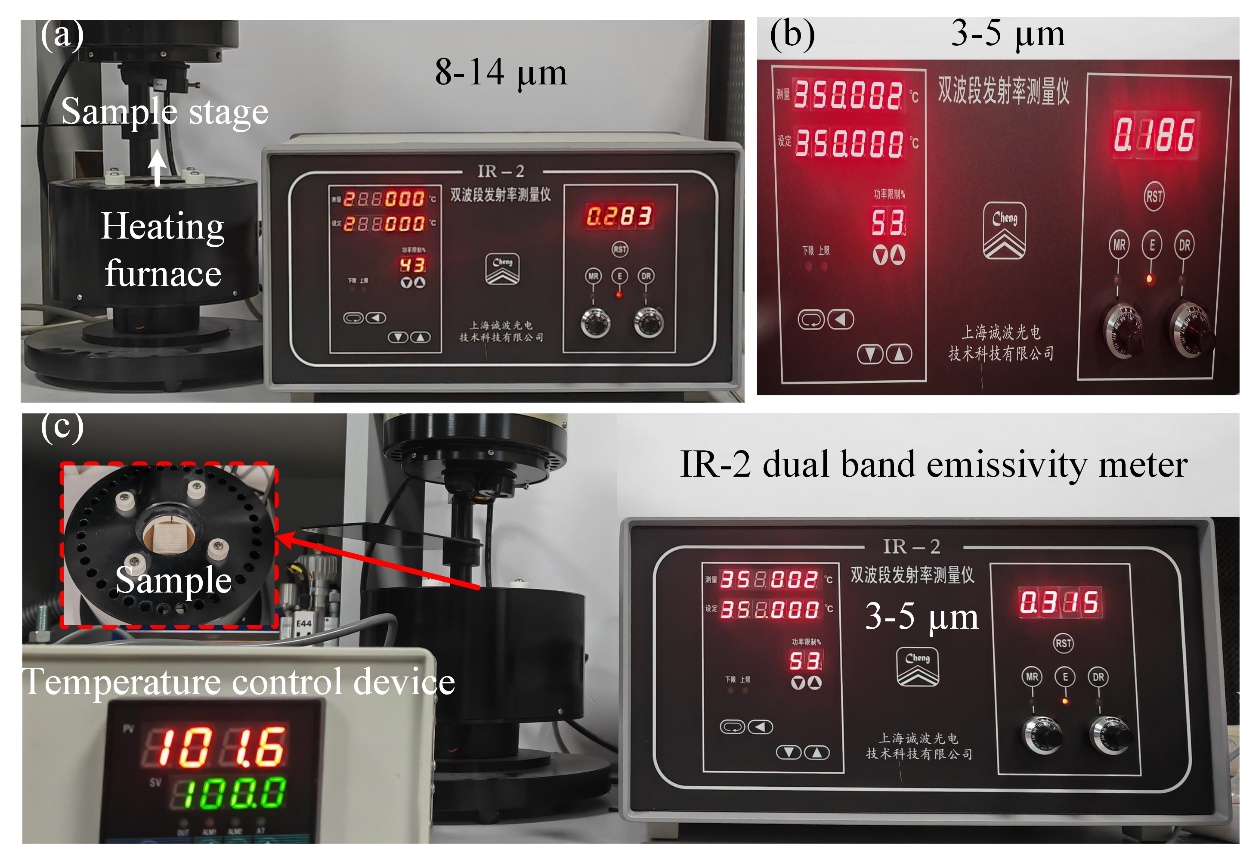


**Figure S10** IR measurement setup and tested IR emissivity at (a) 8–14 μm and 3–5 μm under (b) 27℃ and (c) 100 ℃.
